# Sexual behavior of migrant workers in Shanghai, China

**DOI:** 10.1186/s12889-015-2385-y

**Published:** 2015-10-17

**Authors:** Wei Dai, Jian Gao, Jian Gong, Xiuping Xia, Hua Yang, Yao Shen, Jie Gu, Tianhao Wang, Yao Liu, Jing Zhou, Zhiping Shen, Zhushan Zhu, Zhigang Pan

**Affiliations:** Department of General Practice, Zhongshan Hospital of Fudan University, Shanghai, China; Nutrition Department, Zhongshan Hospital of Fudan University, Shanghai, China; Huangdu Community Health Service Center, Jiading, Shanghai, China

## Abstract

**Background:**

Rapid urbanization of China has resulted in significant domestic migration. The purpose of the present study was to survey the sexual behavior of migrant workers in Shanghai and determine the risk factors for unprotected sex.

**Methods:**

A cross-sectional study of the sexual behavior of 5996 migrant workers was conducted in 7 administrative regions of Shanghai in 2012 from August to October. A self-administered questionnaire was used to collect data.

**Results:**

Five thousand seven hundred seventy two out of the 5996 migrants enrolled into the present study were primarily young adults aged 34.3 ± 10.6 years. Of them, 73.5 % were married, 51.1 % graduated from junior high school, 46.0 % earned 1500–2500 yuan (RMB) monthly. The majority (82.3 %) of the migrants engaged in sexual behavior, and 58.0 % did not use condoms in sexual intercourse. Some of the participants (15.2 %) had casual extramarital partners within the previous 12 months; among them, 76.2 % never or only occasionally used condoms. The results of the multivariate logistic regression analysis suggested that condom use was associated with age, occupation, monthly income, education, and housing conditions. Having temporary sexual partners was significantly associated with several factors such as unmarried (*OR*: 0.47, 95 % CI: 0.38–0.57), working at domestic (*OR*: 1.65,95 % CI: 1.17–2.34), working at wholesale/retail(*OR*: 1.65, 95 % CI: 1.13–2.13), and male migrants (*OR: *2.37, 95 % CI: 1.96–2.85), but not with other factors such as age, monthly income, or education. Having casual extramarital partners was significantly associated with female migrants working at domestic *(OR*: 1.89, 95 % CI: 1.09–3.28), unmarried male migrants (*OR*: 0.51, 95 % CI: 0.36–0.74).

**Conclusion:**

Closer attention should be paid to sexual health education among migrant workers, especially women and those working in domestic and wholesale/retail occupations. The use of condoms should be promoted for older (>35 y), low-income, and less-educated individuals.

## Background

The recent, rapid urbanization of China has resulted in significant domestic migration. Many young adults have migrated from rural to urban areas, especially to large cities, to seek employment opportunities with higher salaries. The economic and social developments of the country are a direct result of their contributions, especially with regard to the construction and maintenance of the cities. Data from the National Bureau of Statistics of China (http://www.stats.gov.cn/tjsj/tjgb/ndtjgb/qgndtjgb/201302/t20130221_30027.html) indicate that there are 160 ~ 170 million rural migrant workers working in cities, with an annual growth rate of 6–8 million in the recent past 5 years. According to the Sixth Demographic Census of China [1], the number of resident migrants in Shanghai (living in Shanghai more than 6 months, with residence permits) increased 8-fold from 1988 to 2012, with an even-faster increasing rate in recent years. Most of them, some 7.03 million were engaged in various industrial settings.

The large number of migrant workers in urban areas has had a great effect on various aspects of society. Because migrant workers often travel from place to place with constant changes in living conditions, most are separated from their spouses for long periods. Most of the migrants are young adults, and sexual behavior that is normal to their place of origin often changes upon moving. When migrants leave their familiar environment, the anonymity may increase risky sexual activities such as having multiple casual sexual partners, engaging in sex with commercial sex workers, and alcohol abuse [2]. It was reported that nearly 80 % of total HIV/AIDS cases in China are found among the domestic migrants [3]. The mode of HIV transmission has changed since several years ago. In 2011, the proportions of HIV infection through heterosexual transmission and homosexual transmission reached 46.5 % and 17.4 %, respectively. Since heterosexual transmission has already become the main mode of transmission in China [4], HIV infections and sexually transmitted infections are quickly spreading from the high-risk groups to the general population [5]. As a special group, the migrant workers have been thought to have a higher level of sexual risk behavior than the rural population [6].

Many scholars have studied the sexual behaviors in migrant workers in different cities in China [7–9], but usually describe only 1–3 local areas or special industries (such as construction and entertainment) or commercial sex workers [10–12]. Studies that concern condom use [13] or the commercialization of sexual relations are limited in terms of sampling strategies [14–16]. Data from some studies indicate that condom use among male migrants remains extremely limited [17, 18]. However, there is a lack of study covering the whole crowd of the migrants.

In the present study, we conducted a survey among migrants in Shanghai in order to identify the characteristics of this population, estimate the status of condom use, and determine the risk factors associated with casual extramarital sex. The focus of the investigation was on the working class of adult migrants, in order to help develop effective intervention approaches for preventing sexually transmitted diseases and improve the lifestyle and living conditions of these people. Although there were certain limitations in the sampling methodology, we attempted to represent the basic situation of adult migrant workers by enrolling migrants from different industries, districts, and age groups; most of them were manual and industrial laborers.

## Methods

In this cross-sectional study, we employed a proportionally stratified multistage cluster random sampling procedure to recruit participants. The inclusion criteria were as follows: 1. had lived in Shanghai for at least 6 months; 2. aged 18–65 years; 3. not registered as a Shanghai resident; and 4.were mainly engaged in a working class profession. The statistically adequate sample size was calculated to be 5800, after considering the prevalence of smoking, alcohol consumption, nonuse of condoms, and mental disorders (since our study was one part of a larger survey of the migrants in Shanghai which included the data on smoking, alcohol consumption, and mental disorders), and a 10 % possible questionnaire failure rate.

**Fig. 1:**
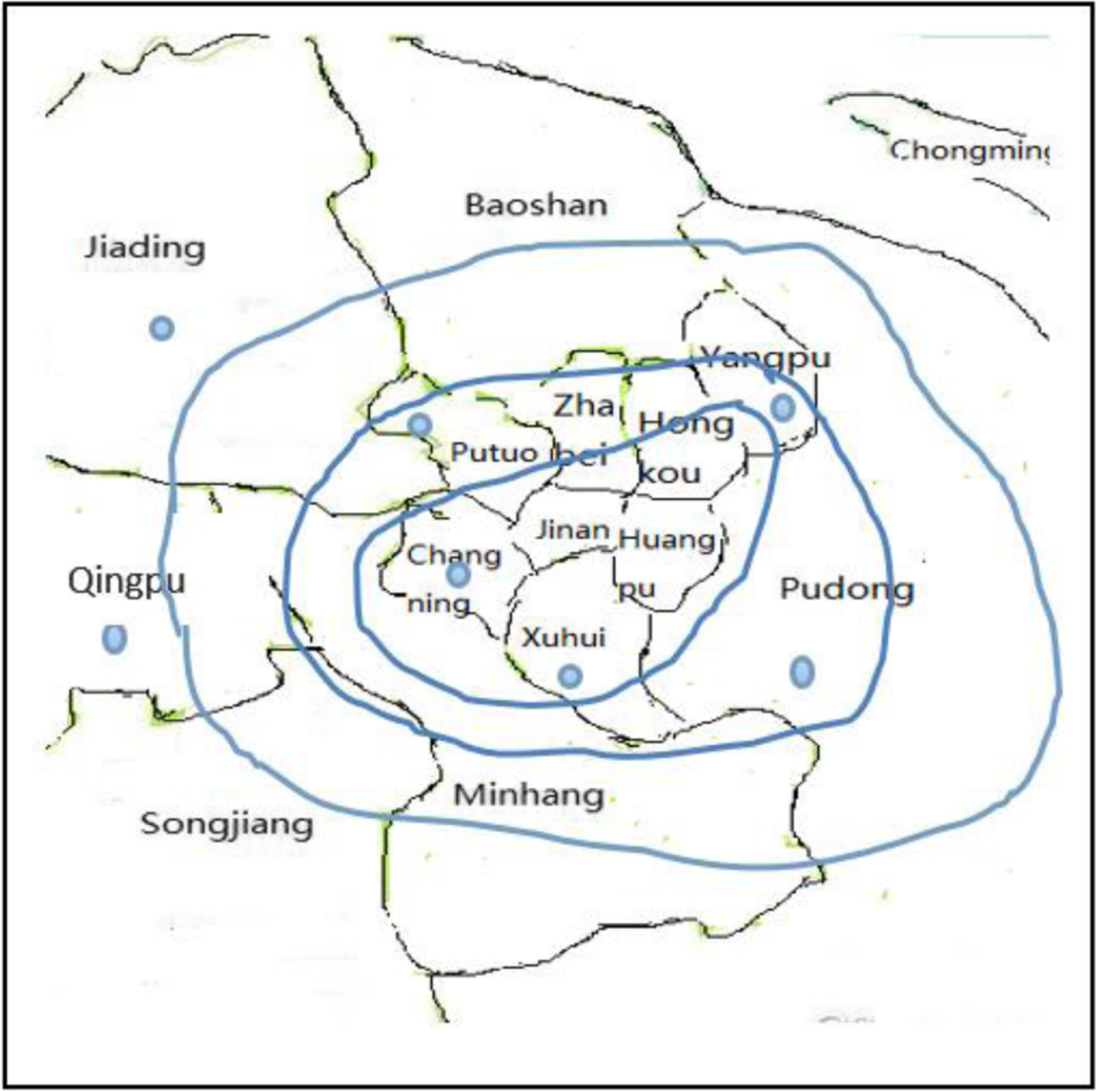
Distribution of the survey sites in Shanghai. The blue points are our survey sites. Three curves represent 3 rings of Shanghai city, from the inside out: the inner, the central, and the outer ring, represent the central, surrounding, and suburban joint areas, respectively

The sample selection process was followed by a three-stage stratified sampling strategy. First, we selected 7 districts in Shanghai which were located in the central (Xuhui and Changning), surrounding (Putuo, Yangpu, and Pudong), and suburban joint areas (Jiading and Qingpu; Fig. 1). Second, one community was randomly selected from each of the 7 districts. Third, the sample subjects were recruited using the quota-sampling procedure from 6 occupational clusters (manufacture, construction, accommodation/catering, domestic service, wholesale/retail, and entertainment) according to the employment of migrants reported by the Shanghai Statistics Bureau in 2012 (http://www.stats-sh.gov.cn/data/toTjnj.xhtml?y=2012). The six occupational clusters were selected because they accounted for approximately 83.7 % of the migrants in East China. Then we calculated the number of participants in each occupational cluster according to the distribution of the migrants by occupational clusters. After the permission of the employers to conduct the survey, we classified all the eligible workplaces in which there were more than 50 % of employees were migrants in three strata based on the number of employees: large, ≥500; moderate, 100–500; small, ≤100. We randomly sampled no more than 200 migrants in each large workplace, no more than 150 migrants in each moderately sized workplace, and all migrants in each small-sized workplaces from the employees lists until the desired sample number for the entire study was reached.

The survey was conducted between August and October 2012. Of 5996 migrants who volunteered to participate in the study, 5772 (96.3 %) (the sample size ranging from 491 to 1070 among 7 districts) completed a face-to-face interview (Table 1).

**Table 1:** Characteristics of participants

Variable	Total (*n* = 5772)	Percent
Gender		
Male	2929	50.9
Women	2833	49.1
Occupation		
Manufacture	2651	45.9
Construction	813	14.1
Accommodation /Catering	399	6.9
Domestic service	615	10.7
Wholesale/Retail	661	11.5
Entertainment	633	11.0
Marital status		
Married	4243	73.5
Unmarried	1529	26.5
Education		
Primary school or below	1058	18.3
Junior high school	2952	51.1
High school or above	1762	30.9
Monthly income (RMB: yuan)		
< 1500	518	9.0
1500–2500	2658	46.0
2500–3500	1780	30.8
> 3500	816	14.1
Housing		
Dormitory	1647	28.5
Renting with friends	661	11.5
Renting with family	2428	42.1
Renting alone	784	13.6
Self-buying	252	4.4
Children (yes/no)		
None	1707	29.6
Yes, in Shanghai ≥ 6 months	1611	27.9
Yes, in Shanghai < 6 months	339	5.9
Yes, but not in Shanghai	2112	36.6
Not answer	3	0.1

A structured questionnaire, which was designed by the authors, primarily based on the scales used in China’s National HIV Surveillance Surveys [19], was used for the individual interview. The questionnaire included information on participants’ social and demographic characteristics and sexual behavior. Demographic information included age, gender, marital status, education, number of children, monthly income, industry of occupation, and housing. Questions regarding sexual behavior included condom use (in the past year; marital and extramarital), casual extramarital sex partners in the past year, symptoms related to sexually transmitted diseases, and use of medicine. In our study, sexual behavior was only defined as sex (heterosexuality by vagina), and casual extramarital sex was defined as having sexual intercourse with another person without pay or plan for a long relationship.

The Ethics Review Committee of Zhongshan Hospital, Fudan University, Shanghai, China approved the study design and the questionnaire (Approval No. B2013-138). Each of the participants provided written informed consent before participating in this survey.

### Data analysis and quality control

Before the survey was conducted, the research staff (about 15–20 in each district) were trained in accordance with the unified guideline for the study. The questions and their arrangement were adjusted based on the results of a pilot survey conducted with approximately 350 migrant workers (about 50 in each district). To ensure privacy during the survey, one investigator alone interviewed one migrant worker in a private room at a given time (after the permission of the employees, we usually conducted the survey during the participants’ rest time). For sensitive questions, the subjects provided answers without interference from the survey staff, except for interpretation of the specific terms in the questionnaire. The answers to the questionnaire were checked by senior investigators to ensure the completeness of the survey and entered into a database (Microsoft Excel) by designated personnel who were blind to the field survey.

The statistical analyses were performed using SPSS version19.0 (IBM, Chicago, IL, USA). The social and demographic characteristics, migratory history, condom use and temporary sexual partner status were presented as the frequency distributions, percentage, mean, and standard deviation. The Chi-square test was performed to examine the associations between the sexual behavior (condom use and casual extramarital partner) and the socio-demographic characteristics, working and living conditions. Multivariate analysis with logistic regression model was applied to identify independent variables associated with condom use and temporary sexual partner, from which adjusted odds (*OR*) and 95 % confidence intervals(CI) were calculated. P-value of less than 0.05 (2-tailed) was considered statistically significant.

## Results

### Demographic characteristics of participants

A total of 5772 valid questionnaires were obtained; 2833 (49.1 %) from women and 2929 (50.9 %) from men (Table 1). The participants’ age ranged from 18 to 65 years old (34.3 ± 10.6 years). 4243 participants (73.5 %) were married, 1388 were single (24.0 %), 88 were in premarital cohabitation (1.5 %), and 53 were divorced or widowed (0.9 %). In terms of educational levels, 2952 (51.1 %) were at the middle school level and 1762 (30.6 %) were at high school level or above. Regarding monthly income, nearly half (46.0 %) of the participants earned 1500–2500 yuan (RMB,100 RMB ≈ 16USD), 30.8 % earned 2500–3500 yuan (RMB), 14.1 % earned above 3500 yuan (RMB), and 9.0 % earned below 1500 yuan (RMB). The average duration of migration was 87.9 ± 80.2 months, and the average time in residence in Shanghai was 66.4 ± 69.4 months. Almost 50.5 % of the participants reported having migrated to only one city, while 8 % reported having migrated to more than five cities.

### Sexual behavior and condom use of the participants

For the 5772 migrant workers surveyed, 4750 (82.3 %) were sexually active, including marital and extramarital sex, while 1017 (17.6 %) were celibate (0.1 % did not answer the question). Of them, 4007 (84.4 %) were married, 614 (12.9 %) were single, 80 (1.7 %) were in premarital cohabitation, and 49 (1.0 %) were divorced or widowed; 723 (15.2 %) had sex with a casual extramarital partner in the past year, including 225 women (31.1 %) and 498 men (68.9 %). 1.3 % did not answer the question concerning casual extramarital partners.

Among the 4750 migrant workers who were sexually active, 58.0 % never used a condom in their sexual intercourse and 30.8 % used them occasionally . Of the 723 migrant workers who had a casual extramarital partner in the past year, 76.2 % either never or only occasionally used a condom.

### The risk factors of unprotected sex and casual extramarital partners in migrant workers

In univariate analyses, the primary outcome, condom use during sexual intercourse, showed significant associations with gender, age, level of education, monthly income, housing, and industry of occupation (Table 2). No statistical association was found between condom use and marital status or whether had children. Sexual relations with a casual extramarital partner were not associated with age, whether had children, or monthly income (Table 2).

**Table 2:** Sexual behavior (Condom use and casual extramarital partner) in migrant workers

Variable	Total	Condom use				Casual extramarital partner			
	(*n*)	*n*	%	*χ* ^2^	*P*_*1*_	*n*	%	*χ* ^2^	*P*_*2*_
Gender									
Male	2472	1040	42.1	6.60	0.01	498	20.1	65.46	<0.01
Female	2278	955	41.9			225	9.9		
Age, y									
18–34	2276	1164	51.1	29.92	<0.01	370	16.3	2.73	0.10
35–65	2474	799	32.2			353	14.3		
Marital status									
Married	4007	1528	38.1	2.80	0.10	505	12.6	18.34	<0.01
Unmarried	743	467	62.9			218	29.3		
Education									
Primary school or below	969	254	26.2	54.59	<0.01	116	12.0	9.66	0.01
Junior high school	2454	1031	42.0			393	16.0		
Senior high school or above	1327	710	53.5			214	16.1		
Monthly income (RMB: yuan)									
< 2500	2552	958	37.5	10.45	<0.01	324	12.7	1.84	0.18
≥ 2500	2198	1037	47.2			399	18.2		
Housing									
Dormitory	1218	514	42.2	15.57	<0.01	217	17.8	14.57	0.01
Renting with friends	488	276	56.6			118	24.2		
Renting with family	2183	799	36.6			232	10.6		
Renting alone	626	309	49.4			129	20.6		
Self-buying	235	97	41.3			27	11.5		
Children (yes/no)									
Yes, but not in Shanghai	1970	748	38.0	7.27	0.03	272	13.8	0.70	0.71
Yes, in Shanghai	1853	655	35.3			216	11.7		
No children	927	560	60.4			235	25.4		
Occupation									
Manufacture	2166	950	43.9	56.06	<0.01	340	15.7	55.63	<0.01
Construction	704	266	37.8			117	16.6		
Accommodation/catering	298	137	46.0			38	12.8		
Domestic service	538	148	27.5			41	7.6		
Wholesale/retail	585	178	30.4			51	8.7		
Entertainment	459	316	68.8			136	29.6		

The multivariate analysis showed that condom use was associated with age, educational attainment, industry of occupation, monthly income, and housing (Table 3). The probability of condom use for migrant workers 35–65 years old was significantly less than for those aged 18–34 years (*OR*: 0.62, 95 % CI: 0.54–0.70). The condom use rate was significantly lower in other industries but higher in the entertainment industry (*OR*: 2.03, 95 % CI: 1.62–2.54). Condom usage was positively associated with educational level, with more highly educated participants being more likely to use condoms (Table 3). Compared to those whose highest educational level was primary school or low, those with more years of education were more likely to use condoms (*OR*: 1.58 cf. 2.14). The migrants with higher incomes were more likely to use condoms than those with lower incomes (*OR*: 1.18, 95 % CI: 1.04–1.34).

**Table 3:** Multivariate analysis of condom use and casual extramarital partner within the past year in migrant workers

	Condom use, *OR* (95 % CI)	Casual extramarital partner, *OR* (95 % CI)
Gender	NS	
Male		1
Female		2.37 (1.96–2.85)
Age, y		NS
18–34	1	
35–65	0.62 (0.54–0.70)	
Marital status	NS	
Married		1
Unmarried		0.47 (0.38–0.57)
Education		NS
Primary school or below	1	
Junior high school	1.58 (1.33–1.88)	
High school or above	2.14 (1.76–2.61)	
Occupation		
Manufacture	1	1
Construction	0.80 (0.65–0.98)	1.12 (0.88–1.42)
Accommodation/catering	0.99 (0.77–1.27)	1.30 (0.89–1.88)
Domestic service	0.65 (0.52–0.81)	1.65 (1.17–2.34)
Wholesale/retail	0.72 (0.58–0.88)	1.56 (1.13–2.13)
Entertainment	2.03 (1.62–2.54)	0.46 (0.36–0.60)
Monthly income (yuan)		NS
< 2500	1	
≥ 2500	1.18 (1.04–1.34)	
Housing		NS
Dormitory	1	
Renting with friends	1.28 (1.01–1.61)	
Renting with family	0.78 (0.66–0.92)	
Renting alone	1.07 (0.86–1.33)	
Self-buying	0.90 (0.67–1.23)	

Of the 4750 participants, 62 (1.3 %) did not answer the question about having a casual extramarital partner in the past year (Table 3). Univariate analysis showed that having a casual extramarital partner was significantly associated with gender, marital status, level of education, occupation, and housing (*P* < 0.01).

Multivariate analysis showed that having casual extramarital partners was not related to age, education, monthly income, or housing, but closely related to marriage, occupation, and gender (Table 3). The migrants who engaged in domestic service and wholesale/retail were more likely to have casual extramarital sex (*OR*: 1.65, 95 % CI: 1.17–2.34; *OR*: 1.56, 95 % CI: 1.13–2.13, respectively)

Women appeared more likely to have casual extramarital partners than men (Table 4). The multivariate analysis indicated that the possibility of having casual extramarital partner in women was associated with marital status, education, housing, and industry type of occupation (*P* < 0.05), while the same figure for men was associated with marital status and occupation (Table 4, *P* < 0.05).

**Table 4:** Multivariate analysis of migrant workers who had sexual behavior with casual extramarital partner within the past year by gender

Variable	Model 1 (Female)				Model 1 (Male)			
	Casual extramarital partner	Non- Casual extramarital partner	*P*	*OR* (95 % CI)	Casual extramarital partner	Non- Casual extramarital partner	*P*	*OR* (95 % CI)
Number of subjects, *n*	225	2028			498	1937		
Age, y								
18–34	153	1058	0.73	1	217	822	0.07	1
35–65	72	970		0.94(0.64–1.37)	281	1115		0.81(0.64–1.02)
Marital status								
Married	141	1820	0.04	1	364	1632	<0.01	1
Unmarried	84	208		0.57(0.33–0.99)	134	305		0.51(0.36–0.74)
Education								
Primary school or below	49	543	0.01	1	160	659	0.11	1
Junior high school	121	1051		2.13(1.29–3.53)	271	978		0.91(0.65–1.27)
High school or above	54	435		1.37(0.91–2.05)	67	300		0.79(0.62–0.99)
Occupation								
Manufacture	67	863	<0.01	1	273	937	0.05	1
Construction	7	73		1.10(0.46–2.63)	110	500		1.40(1.05–1.87)
Accommodation/catering	10	160		1.49(0.74–3.00)	28	97		1.16(0.74–1.82)
Domestic service	21	384		1.89(1.09–3.28)	20	109		1.66(1.01–2.75)
Wholesale/retail	18	348		1.48(0.85–2.59)	33	180		1.53(1.02–2.29)
Entertainment	102	200		0.27(0.17–0.41)	34	114		1.31(0.85–2.01)
Monthly income (RMB:yuan)								
< 2500	113	1405	0.38	1	211	792	0.97	1
≥ 2500	112	623		0.86(0.61–1.21)	287	1145		1.00(0.82–1.24)
Housing								
Dormitory	40	317	0.03	1	177	662	0.30	1
Renting with friends	82	1229		1.67(1.06–2.65)	150	703		1.26(0.94–1.68)
Renting with family	60	165		0.84(0.50–1.42)	58	197		1.04(0.73–1.48)
Renting alone	35	207		1.26(0.73–2.19)	94	284		0.91(0.67–1.25)
Self-buying	8	110		1.02(0.43–2.40)	19	91		1.27(0.73–2.22)
Children (yes/no)								
Yes, but not in Shanghai	72	821	0.38	1	200	855	0.93	1
Yes, in Shanghai	58	900		1.10(0.75–1.62)	158	707		1.10(0.75–1.62)
No children	95	307		0.73(0.43–1.24)	140	375		0.93(0.64–1.36)

## Discussion

According to the published literatures, risk sexual behavior was defined as having multiple sex partners, paying for sex, and homogeneity sex, etc. As we all know, heterosexual transmission has become the main mode of HIV/STD transmission in China. Yang et al. [20] have found that about 40.0 % of migrants fail to understand that use of condoms decreases the risk of HIV infection. Migrants who have engaged in sex with commercial sex workers have better HIV knowledge than migrants who have never paid for sex. The present study was one part of a large survey of the migrants in Shanghai. It took the participants about 35 min to fill in the questionnaire. We only selected the condom use and casual noncommercial extramarital sex as indices to assess the sexual behavior of migrants. To our knowledge, this study was the first to document the sexual behavior and condom use in China and to assess the possible factors for unprotected sex among migrant workers.

We found a low proportion of condom use among both male and female migrant workers. In our study, 58.0 % of participants (57.9 % and 58.1 % for males and females respectively) never used a condom in their sexual intercourse and 76.2 % either never or only occasionally used a condom with their casual extramarital sex. Studies in India (25.0 %), South Africa (33.0 %) and Croatia (44.7 %) have revealed that condom use is less practiced among migrant workers having sexual contact with any casual or commercial sexual partners [21–23]. Wang et al. [24] have found that there are 73.7 % unmarried male migrants in Shanghai who had sexual intercourse had not used condoms in their last sexual intercourse and 50.6 % reported never or occasionally used a condom with their casual extramarital sex. The condom use in general group in the present study was higher than previously reported by other studies, but lower in the casual extramarital sex group than others. As Wang *et al*. [24] reported, participants who perceived themselves to be at low risk of HIV infection were more likely to have non-regular sexual partners than those at a higher risk., The education of condom use in migrants is necessary especially among those who had casual extramarital sex.

We also found the association between condom use with age, education, occupation, monthly income, and housing. It seemed the younger migrants who earn more and had higher education level had higher possibility of condom use. It could be explained that the younger who were better educated had more knowledge of the HIV/STD. But several Chinese studies thought that the primary reason given for condom use among migrants is contraception instead of disease prevention [25, 26]. We also found that the migrants who engaged in entertainment had higher possibility of condom use (*OR*: 2.03, 95 % CI: 1.62–2.54, for manufacture). In the present study, the migrants engaged in entertainment included those who worked at bath houses, night clubs, beauty salons, and hair salons; it could be explained by the effect of the education in high-risk groups of HIV/STD. Condom use is one of the most effective means of preventing infection from the sexually transmitted diseases [27]. It was reported that consistent and correct condom use could reduce the risk of HIV infection by approximately 69 % [27, 28].

The present study also found associations between the casual extramarital sex and gender, marital status, and industry of employment. Unmarried migrant workers had fewer encounters with a casual extramarital partner than their married counterparts (*OR*: 0.47, 95 % CI 0.38–0.57), which was different from what was observed by Wang et al. [24]. This difference may be related to differences in the target populations of these studies. In the present study, compared to migrant workers engaged in manufacture, those in entertainment had fewer casual extramarital partners, which is contrary to previously thought. This may be due to the definition for casual extramarital sex partner in our study. It is believed that the entertainment industry is where most commercial sexual behaviors exist. We also found that those engaged in domestic service, which had a lower rate of condom use (27.5 %), had more possibility of having casual extramarital sex than those engaged in manufacture. This finding is beyond our expectation. It also suggested that we should not only focus on high risk groups but also pay attention to low risk ones when carrying out health education of sexually transmitted diseases, as what mentioned in the previous paragraph: those who had low risk of HIV infection were more likely to report non-regular sexual partners than those having a higher risk perception.

Another surprising finding of the present study was that female migrants had more possibility of having casual extramarital sex than male ones (*OR*: 2.37, 95 % CI: 1.96–2.85). Fang et al. [29] studied female sex workers but did not mention their non-commercial sexual behaviors. The further multivariate analysis indicated that those female migrants who were married, engaging in domestic service, renting house with friends were more likely to have casual extramarital sex (*p* < 0.05). While for male migrants marital status was the major factor (*p* < 0.01). Unmarried men were less likely (*OR*: 0.51, 95 % CI: 0.36–0.74) to have casual extramarital sex than married men, which is inconsistent with earlier research [24, 29]. One reason may be that we surveyed the general population of migrants, and the sexual encounters with casual extramarital partners that were considered were non-commercial only. Another reason should be the special social-psychological characteristics of migrants. Migration is a primary cause of behavior change. When the migrants were away from their spouses, families and homes, they were forced into physically demanding jobs and poor housing and living conditions, which may put them at risk of HIV infection [30, 31]. The studies in South Africa (31.4 %), North Carolina (46.0 %), and California (30.0 %) indicated that migrant workers living apart from their wives were likely to engage in higher rates of multiple and commercial sex [30]. Though in our study,we did not care about the commercial sex, for migrants, the sense of emptiness and anonymity of being a foreigner might increase the risky sexual activities. As a pity, we did not do the further research about the difference of psychological characteristic between female and male migrants.

This study was only one part of a total study on migrant workers in Shanghai, and has some limitations. First of all, because of the cross-sectional design and non-random, quato sampling selection of participants, we cannot draw conclusions of the causal relationship. Besides, the self-reported data of the migrants may cause the recall and social desirability bias. Unfortunately, we had no laboratory test data to confirm associations among condom usage, casual extramarital sex, and sexually transmitted diseases [32, 33]. The study was limited to a single city, and although Shanghai has one of the largest migrant populations in China, it is hard to draw a general conclusion on the sexual behavior of migrant workers in all of China. And because of limits of the time and fund, we also did not include other sexual behaviors such as homosexual intercourse and engaging multiple sexual partners.

## Conclusions

As a result of poor condom promotion, education, and utilization efforts [34], the risk is enhanced by the low frequency of consistent condom use among returnee migrants having sexual contact with their spouses and regular sexual partners. In addition, migrant workers are less willing to use condoms because of connotations of multiple partnerships [35]. Our results are a reminder that we should support safe-sex education programs in those industries with low condom usage and a high possibility of casual partners. It is suggested that more attention should be paid to sexual health education given to women, and those employed in domestic service or wholesale and retail sales [36]. Condom use should be encouraged, especially in older, low-income, and less-educated populations. In addition, the appropriate focus of sexual health education may differ between men and women migrants. Further studies are needed to explain why women migrants in domestic service are more likely to have a casual extramarital partner than men and the different psychological characteristic between female and male migrants.

## Abbreviations

HIV: Human immunodeficiency virus
SPSS: the Statistical Program for Social Sciences
RMB: Renminbi
USD: United States Dollar
OR: Odds ratio
CI: Confidence interval
NS: No significance
