# Erratum to: Sexual behavior of migrant workers in Shanghai, China

**DOI:** 10.1186/s12889-015-2487-6

**Published:** 2016-09-30

**Authors:** Wei Dai, Jian Gao, Jian Gong, Xiuping Xia, Hua Yang, Yao Shen, Jie Gu, Tianhao Wang, Yao Liu, Jing Zhou, Zhiping Shen, Shanzhu Zhu, Zhigang Pan

**Affiliations:** 1 Department of General Practice, Zhongshan Hospital of Fudan University, Shanghai, China; 2 Nutrition Department, Zhongshan Hospital of Fudan University, Shanghai, China; 3 Huangdu Community Health Service Center, Jiading, Shanghai, China

## Erratum

After publication of the original paper [1] it came to our attention that the name of author Shanzhu Zhu has accidentally been displayed as ‘Zhushan Zhu’. Shanzhu Zhu is the correct spelling.
